# A new framework for the labeling of odors in the Sniffin’ Sticks odor identification test: relevance of familiarity and test–retest assessment

**DOI:** 10.1007/s00405-026-10379-6

**Published:** 2026-06-15

**Authors:** Agnieszka Sabiniewicz, Nadine Brühl, Antje Haehner, Thomas Hummel

**Affiliations:** Department of Otorhinolaryngology, Smell and Taste Clinic, TU Dresden, Fetscherstrasse 74, 01307 Dresden, Germany

**Keywords:** Olfaction, Smell, Sniffin’ Sticks, Odor identification test

## Abstract

**Purpose:**

The Sniffin’ Sticks test is among the most commonly used methods of olfactory assessment and has been successfully adapted across various countries. However, to date, no study has examined cultural changes over time, within the same country. The present research is the first to explore the adaptation of odor labels to contemporary contexts.

**Methods:**

A total of 83 normosmic participants (57 women) aged 18 to 75 years (y) (M = 35.2 y, SD = 13.7) were enrolled in four studies in which we replaced the unfamiliar odor labels with more familiar ones and balanced the frequency of labels used in the entire set of odor labels. Lastly, forty additional healthy participants (28 women) aged from 18 to 61 y (M = 32.8 y, SD = 11.4) were recruited to assess the test–retest reliability of the newly developed version of SS-OIT labeling within a period ranging from 1 to 57 days (M = 14.8 days, SD = 13.2).

**Results:**

We aimed to increase the number of labels that are known by the present population, and hence, we modified 31% of the odor labels. The correlation coefficient of the new SS-OIT test–retest was moderate (*R* = .66 which changed to *R* = .71 after excluding an outlier, *p* < .001), which is typical for 16-item olfactory tests when tried in a normosmic population.

**Conclusion:**

This study introduces a fresh approach to SS-OIT labeling via a careful refinement and validation of an existing test that aligns more closely with evolving cultural trends. We also confirm the suitability of the new SS-OIT for clinical and research assessment.

**Supplementary Information:**

The online version contains supplementary material available at https://doi.org/10.1007/s00405-026-10379-6.

## Introduction

Olfactory tests have gained importance during the SARS-CoV-2 pandemic when used as a clinical indicator of the disease [1, 2]. Among various ways of olfactory assessment, the identification test procedure is the fastest and easiest to follow for both patients and experimenters [3, 4] and hence it is widely popular, also as a biomarker for COVID-19 [5]. However, the indicative role of odor identification is much broader. It ranges from odor perception [e.g., 3] through mood [e.g., 6] to cognitive performance [e.g., 7–9].

Numerous studies have demonstrated that a decrease in odor identification is linked to decreased general cognitive abilities, such as declines in memory, executive function, language, and global cognition scores [e.g., 10, 11]. Early phases of Alzheimer’s disease have significant negative repercussions on odor identification [10], and individuals with olfactory and cognitive impairment are at a potentially higher risk of dementia [12]. Furthermore, impairment in odor identification was reported in depression [6, 13, 14] and, on the opposite, an increase in olfactory function, particularly odor identification, was associated with an improvement of symptoms of depression [15].

Considering the significance of odor identification, it is not surprising that such tests are among the most commonly used in the assessment of odor performance [3, 16–18]. Numerous versions of the identification test have been developed, varying mainly in the number of different odors used. Among these, one of the most widespread is the Sniffin’ Sticks test [3, 19], which uses pen-like odor dispensers. Odor identification (SS-OIT) is available in several versions, varying in terms of the number of odors from 3 [20] to 32 items [21].

It should be carefully considered that performance in odor identification is associated with verbal abilities [22] and cultural context, such that tests need to be specifically adapted for various countries and cultures [e.g., 23, 24]. Also, performance in identification tests can be very sensitive to even minor modifications of the procedure, such as more contrasted distractors that improve the identification test results [25].

But what if the culture changes? Even within the same country, the odor labels commonly known almost 30 years ago, when the Sniffin’ Sticks test was developed [3], might not be recognizable anymore. For example, “green apple” was a prevalent odor used in soaps and washing powders during the 80s and later, but nowadays, young people are not familiar with that scent. Also, turpentine used to be more commonly present, for example, as paint solvent, which is used much less in this form. For that reason, we aimed, in the present study, to carefully refine and validate the original SS-OIT by (a) replacing the unfamiliar odor labels with more familiar ones and (b) balancing the frequency of labels used in the entire set of odor labels.

## Materials & methods

**Fig. 1:**
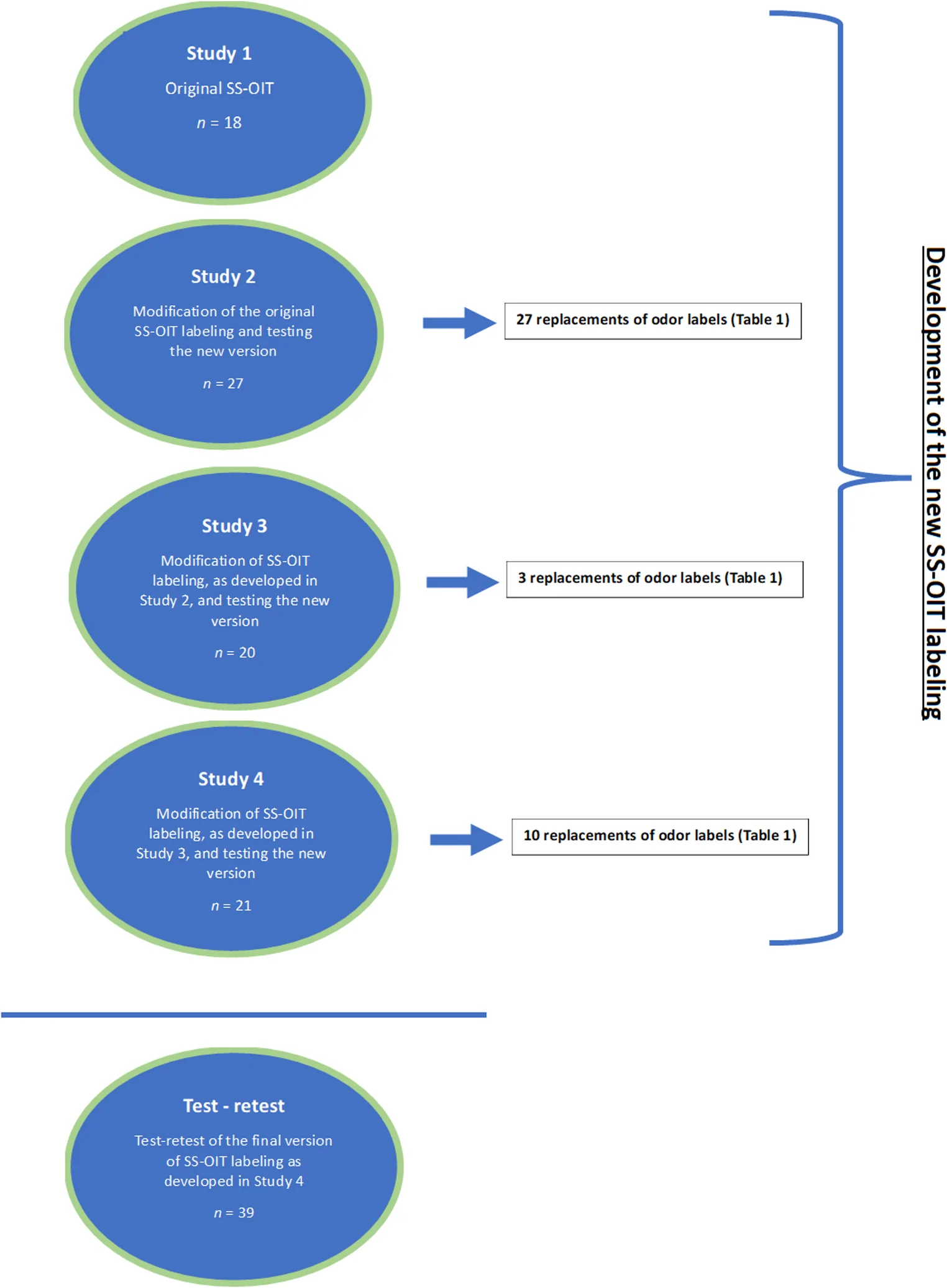
Schematic procedure of the study

**Fig. 2:**
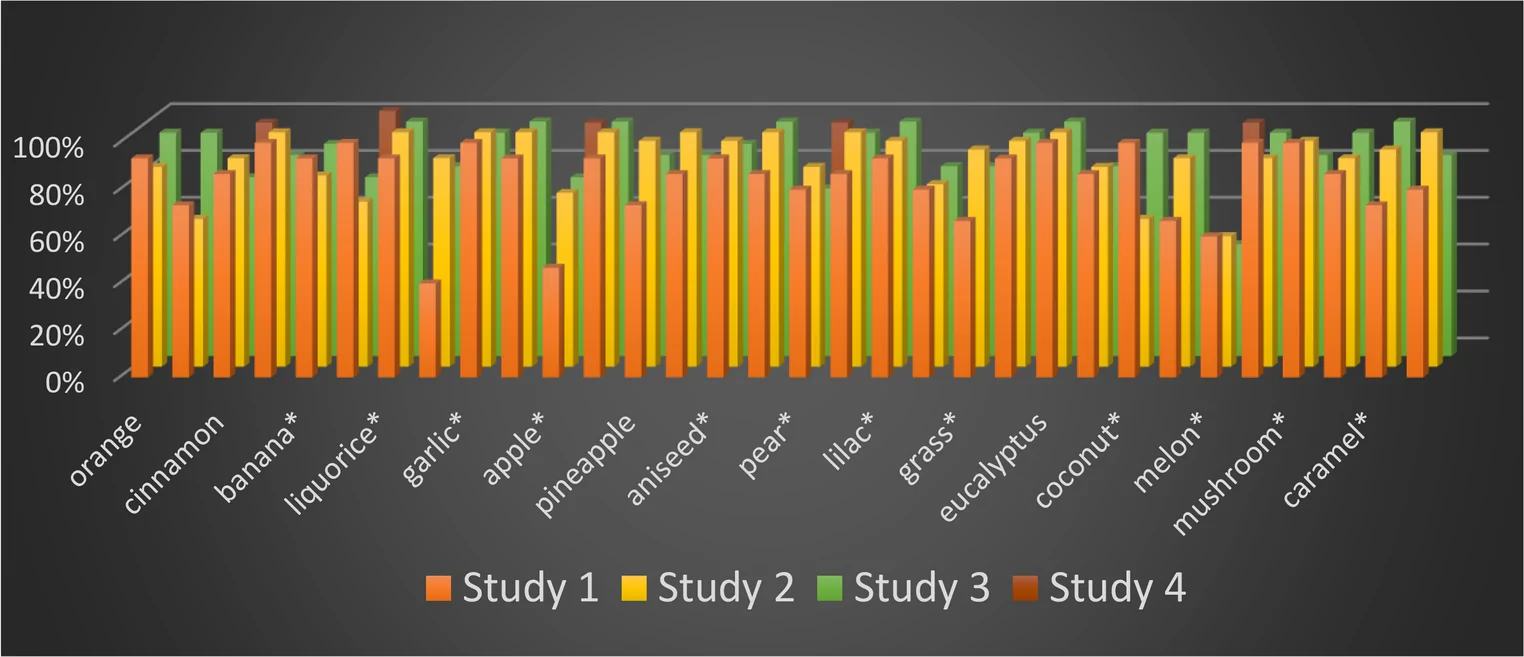
The average identification rate (%) for all four studies

Figure 1 presents a simplified procedure of the study. Firstly, we aimed to develop the new SS-OIT labeling, and hence, we ran four studies, each conducted using a different sample of participants. In Study 1, we conducted the extended version of the Sniffin’ Sticks odor identification test [21]. The criterion of modification was the percentage of correct identification lower than 60%. Throughout the various steps of the following procedure care was taken to balance the frequency of odor labels. In Study 2, we marked and replaced the frequently incorrect or rarely selected options and tested the new version of SS-OIT. The criterion of modification was that the percentage of correct identification should be higher than 70%. We repeated the same procedure in Study 3 and Study 4 to obtain more data. The criterion of modification was a percentage of correct identification of more than 80%. The final list of odor labeling and identification rate of the target labels in the final version of SS-OIT are presented in Supplementary Table 1. Figure 2 shows the average identification rate for all four studies. In all four studies, the presented target odors were rated in terms of intensity (0–10) and pleasantness (-5 to + 5).

Eventually, to assess the reliability of the newly developed version of SS-OIT labeling, in Study 4, a test–retest analysis of its final version was run. A detailed description of the studies is presented below.

### Participants

#### Development of the new SS-OIT labeling

A total of 83 normosmic participants (57 women) aged 18 to 75 years (y) (M = 35.2 y, SD = 13.7) were enrolled for the four studies. More precisely, in Study 1, the extended Sniffin’ Sticks odor identification test (21) was tried out in a sample of 18 participants (11 women aged 18–88 y; M = 39.9 y, SD = 24 and 7 men aged 24–65 y; M = 43.9 y, SD = 16.8). In Study 2, the new version of SS-OIT was tested on a sample of 27 participants (21 women aged 22–75 y; M = 36.5 y, SD = 13.4 and 6 men aged 28–46 y; M = 36.3 y, SD = 8). In Study 3, the modified version of SS-OIT was tested on a sample of 21 participants (17 women aged 19–70 y; M = 35.7 y, SD = 16.3 and 4 men aged 22–54 y; M = 35.5 y, SD = 15.2). In Study 4, the latest version of SS-OIT, as developed in Study 3, was tested on a sample of 21 participants (11 women aged 21–52 y; M = 31.2 y, SD = 8.8 and 10 men aged 20–63 y; M = 34.5 y, SD = 14).

#### Test–retest reliability assessment of the final version of SS-OIT labeling

Forty additional participants (28 women) aged from 18 to 61 y (M = 32.8 y, SD = 11.4) were recruited to assess the test–retest reliability of the newly developed version of SS-OIT labeling within a period of time ranging from 1 to 57 days (M = 14.8 days, SD = 13.2).

Participants were recruited through flyers and word of mouth, patients with olfactory loss were included through the Smell and Taste Clinic at the Department of Otorhinolaryngology of the TU Dresden. The first part of this study comprised a short questionnaire about lifestyle factors (smoking, drinking) or health problems (e.g., head trauma, medication, diabetes) that could potentially affect olfactory function. None of the participants indicated such issues.

To further validate the final version of SS-OIT labeling, we included 40 hyposmic patients (23 women) aged 21 to 85 (y) (M = 56.1, SD = 13.7).

This was a non-interventional psychophysical study. All participants provided written informed consent prior to inclusion in the study. The study complied with the Declaration of Helsinki on Biomedical Research Involving Human Subjects and had been approved by the Ethics Committee at the Medical Faculty of the TU Dresden (application number EK408102018).

### Data analyses

The normality of the data distribution in the final version of SS-OIT was investigated via the Shapiro-Wilk test. Given the nonparametric nature of the distribution, respectively non-parametric tests were employed in further analyses. Specifically, to measure test reliability, we conducted Spearman correlations between the test and retest. Additionally, a Bland-Altman plot [26] was produced to examine the test–retest reliability in more detail. The Welch t-test was used to compare the performance on the final version of the SS-OIT between hyposmic and normosmic participants. Additionally, we conducted a Wilcoxon signed-rank test to compare the performance between the old and new versions of the 16-item SS-OIT in hyposmic patients.

Furthermore, Spearman correlations were employed to explore demographic characteristics such as age and their relation to the performance in odor identification. Wilcoxon-signed rank test was used to compare performance in odor identification tests between genders.

Additionally, General Linear Mixed Model (GLMM) was employed to investigate whether assessed intensity and pleasantness of the presented odors allowed to predict their correct recognition. Odor recognition (correct vs. incorrect) was used as a dependent variable and odor intensity together with odor pleasantness were fixed effects variables. Participants’ IDs was inserted as a random-effects grouping factor.

## Results

### Descriptive statistics

The Shapiro–Wolf test indicated a non-normal distribution of the final version of SS-OIT test scores (Shapiro–Wolf = 0.9, *p* < .001, Median = 31, IQR = 1.3), with the majority of responses skewing to the right (Skewness = -1, SES = 0.4, Kurtosis = 0.5, SEK = 0.7).

### Reliability assessment of the final version of SS-OIT labeling

**Fig. 3:**
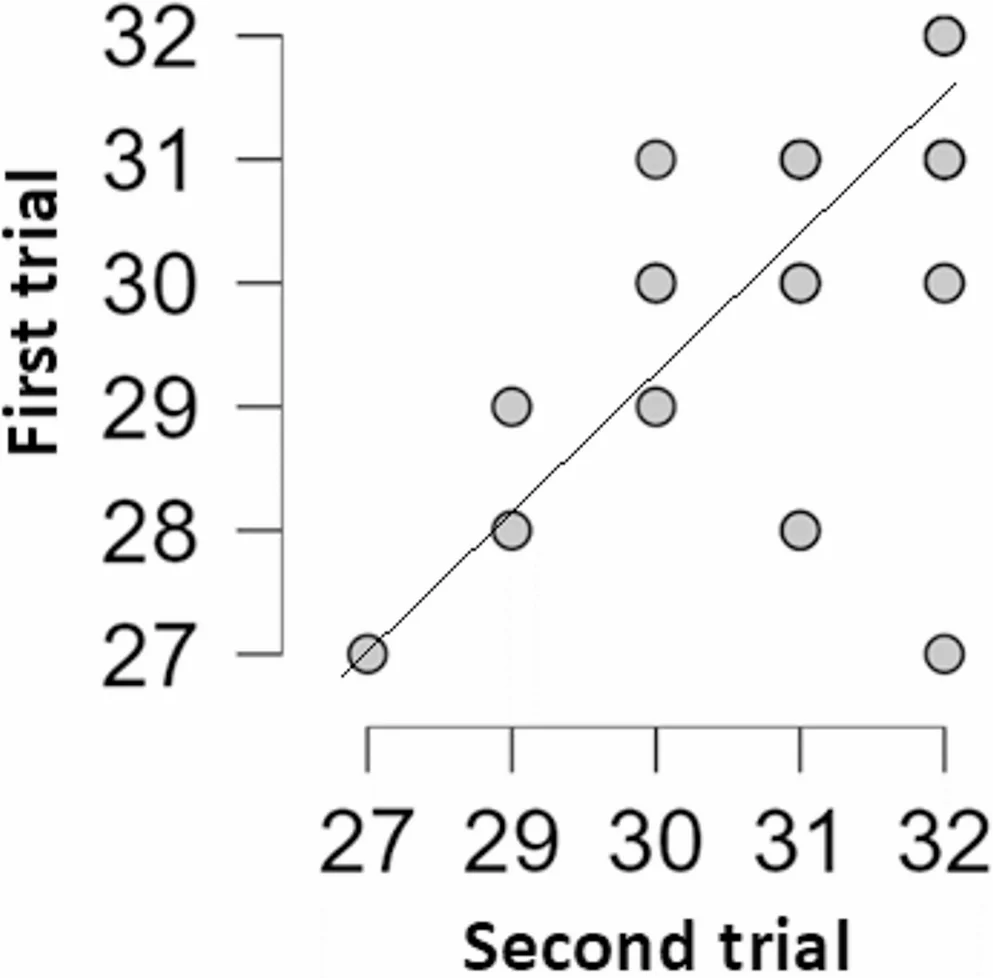
Test–retest reliability of SS-OIT

Spearman correlations indicated moderate reliability (*R* = .66, *p* < .001; Fig. 3). After excluding the outlier with coordinates 27–32, the correlation coefficient was slightly higher (*R* = .71, *p* < .001).

**Fig. 4:**
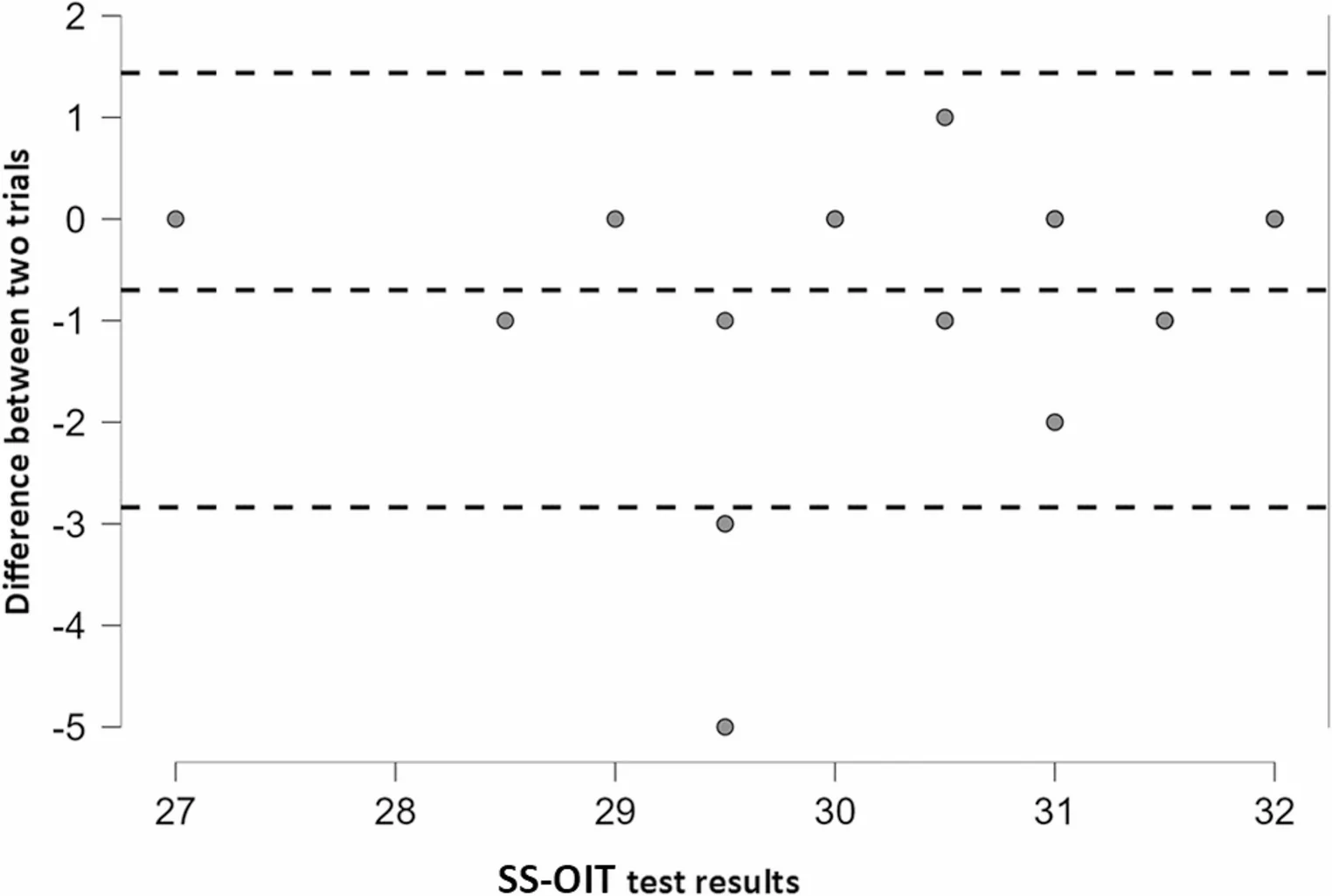
Bland–Altman plot for SS-OIT test–retest

Bland–Altman plot (Fig. 4) showed that only 2 individuals had scores outside of the boundaries of the Bland–Altman plot defined by the difference of the results from the test and retest versus the mean of the two data points. This suggested a good reproducibility of the test results, especially when considering that one of those two scores was very close to the boundaries.

### Comparison of normosmic and hyposmic participants

**Fig. 5:**
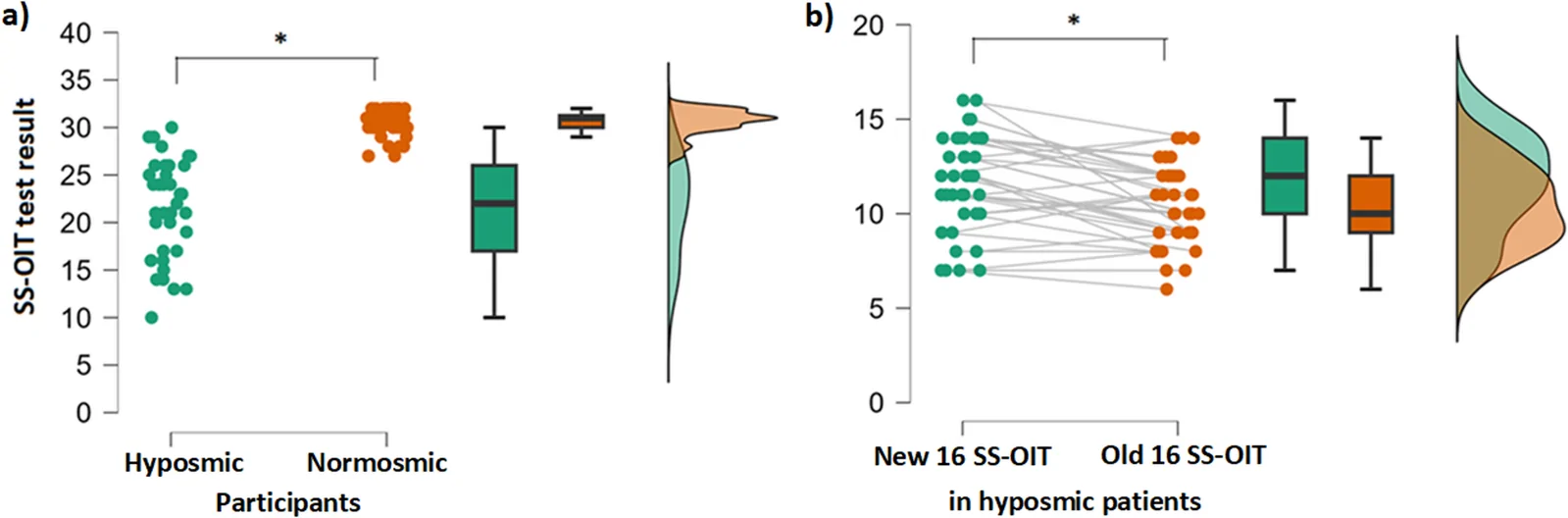
**a**) Welch t-test between the performance in the final SS-OIT of normosmic and hyposmic participants. **b**) Wilcoxon-signed rank test between the performance in the old and new versions of 16-item SS-OIT in hyposmic patients

Normosmic participants significantly outperformed hyposmic ones in the final SS-OIT (t[40.8] = 10.2, *p* < .001, Cohen’s d = 2.4; Fig. 5a). Hyposmic patients performed significantly better in the new version of 16-item SS-OIT compared to the old one (Z = 3.6, *p* < .001, *r* = .71; Fig. 5b).

### Demographic characteristics

Age was negatively and significantly related to SS-OIT performance (*R* = − .42, *p* = .006). No difference was noted between men’s and women’s performance in the test (W = 212.5, *p* = .178).

### Effects of assessed intensity and pleasantness of odors on odor recognition

The GLMM showed no effects of either intensity (x^2^ = 14.7, *p* = .1) or pleasantness (x^2^ = 0.06, *p* = .811) on odor recognition.

## Discussion

The current study, conducted on a large sample of normosmic participants and hyposmic patients, led to a careful refinement and validation of an existing test through the development of a new SS-OIT labeling. We aimed to increase the number of labels that are known by the present population, and hence, we modified 31% of the odor labels. For example, we replaced “turpentine” with “paint thinner” because turpentine, as a label, was poorly recognized some years ago even in normosmic population [27], suggesting that this item is less suitable. Likewise, we exchanged “green apple” with “apple” for similar reasons, in order to improve item suitability. Such change resulted in an increase in recognition rate from 40% to 81%. However, it should be noted that in the case of some odor labels, such as peppermint, lemon, pear, and melon, no increase was found in their identification even after the label modification. This might be related to the distractors in the multiple-choice task [25].

To assess whether the new SS-OIT labeling is suited for daily clinical and research practice, we investigated its statistical properties. The correlation coefficient of the new SS-OIT test–retest was moderate (*R* = .66, which changed to *R* = .71 after excluding an outlier), which is typical for olfactory tests when tried in a healthy, normosmic population, which is relatively homogeneous [28]. The reason for a moderate correlation coefficient might be, to some degree, a certain inherent variability of olfactory testing. Importantly, the differences between test and retest were never larger than two data points. Furthermore, the obtained coefficient did not differ from the one reported in the publication on the original SS-OIT test–retest (*r* = .73 [3]) while the extended 32-item version exhibits a higher test-retest reliability (*r* = .88, *p* < .001 [21]). In this context, it is worth mentioning that while 40 participants took part in the test–retest procedure in the current study, the original publication reported data from 104 participants, which is almost three times more. Hence, the obtained moderate correspondence between the test–retest indicates that the new SS-OIT labeling allows a precise evaluation of olfactory function, as measured via odor identification. Comparison of the normosmic vs. hyposmic participants’ performance in the new SS-OIT is also in line with this conclusion. Still, the present findings should be interpreted with the understanding that moderate reliability is an inherent limitation of olfactory identification based on relatively few items.

In the present study, we also assessed the influence of demographic factors on test performance in order to examine its validity. Older participants scored lower in SS-OIT than younger ones, reflecting a typical decline in olfactory performance [29]. Regarding gender, no differences have been observed, which is in contrast to previous studies [30]. However, it should be considered that an increase in the research sample might lead to some gender-related differences. On the other hand, the present results also show that effects of gender on odor identification are not very strong.

The Shapiro–Wilk test revealed a non-normal distribution of SS-OIT scores. The data showed a moderate negative skew and a leptokurtic pattern, with most participants scoring higher on the test. This type of distribution has also been observed in other olfactory tests commonly used in clinical practice [31, 32]. We conclude that the new, refined and validated SS-OIT, as the original one [3, 21], is suited for the assessment of olfactory function in a clinical context and in research.

The present study is not free from limitations. Specifically, several parts of the study rely on relatively small sub-samples, particularly during the label optimization phases. Hence, the decisions to modify or retain specific odor labels were based on limited data; therefore, the observed improvements in identification rates should be interpreted with caution, as they may partly reflect sample-specific effects rather than stable item properties.

This study introduces a fresh approach to SS-OIT labeling via a careful refinement and validation of an existing test that aligns more closely with evolving cultural trends. We also demonstrate its satisfying reliability and psychometric properties, confirming the suitability of the new SS-OIT as a method for clinical and research assessment.

## Electronic supplementary material

Below is the link to the electronic supplementary material.

Supplementary Material 1

## References

[1] Hannum ME, Ramirez VA, Lipson SJ, Herriman RD, Toskala AK, Lin C, Reed DR (2020) Objective sensory testing methods reveal a higher prevalence of olfactory loss in COVID-19–positive patients compared to subjective methods: A systematic review and meta-analysis. Chem Senses 45:865–874 10.1093/chemse/bjaa064 PMC7543258 33245136

[2] Whitcroft KL, Hummel T (2020) Olfactory dysfunction in COVID-19: diagnosis and management. JAMA 323:2512–2514 10.1001/jama.2020.8391 32432682

[3] Hummel T, Sekinger B, Wolf SR, Pauli E, Kobal G (1997) Sniffin’ Sticks’: olfactory performance assessed by the combined testing of odor identification, odor discrimination and olfactory threshold. Chem Senses 22:39–52 10.1093/chemse/22.1.39 9056084

[4] Landis BN, Hummel T (2006) New evidence for high occurrence of olfactory dysfunctions within the population. Am J Med 119:91–92 10.1016/j.amjmed.2005.07.039 16431204

[5] Moein ST, Hashemian SM, Tabarsi P, Doty RL (2020) Prevalence and reversibility of smell dysfunction measured psychophysically in a cohort of COVID-19 patients. Int Forum Allergy Rhinol 10(10):1127–1135 10.1002/alr.22680 PMC7436559 32761796

[6] Kohli P, Soler ZM, Nguyen SA, Muus JS, Schlosser RJ (2016) The association between olfaction and depression: a systematic review. Chem Senses 41:479–486 10.1093/chemse/bjw061 PMC4918728 27170667

[7] Hedner M, Larsson M, Arnold N, Zucco GM, Hummel T (2010) Cognitive factors in odor detection, odor discrimination, and odor identification tasks. J Clin Exp Neuropsychol 32:1062–1067 10.1080/13803391003683070 20437286

[8] Jacobson PT, Vilarello BJ, Tervo JP, Waring NA, Gudis DA, Goldberg TE, Overdevest JB (2024) Associations between olfactory dysfunction and cognition: a scoping review. J Neurol 271:1170–1203 10.1007/s00415-023-12057-7 PMC11144520 38217708

[9] Challakere Ramaswamy VM, Schofield PW (2022) Olfaction and executive cognitive performance: a systematic review. Front Psychol 13:871391 10.3389/fpsyg.2022.871391 PMC9125097 35615205

[10] Larsson M, Semb H, Winblad B, Amberla K, Wahlund LO, Bäckman L (1999) Odor identification in normal aging and early Alzheimer’s disease: effects of retrieval support. Neuropsychology 13:47 10.1037//0894-4105.13.1.47 10067775

[11] Rezek DL (1987) Olfactory deficits as a neurologic sign in dementia of the Alzheimer type. Arch Neurol 44:1030–1032 10.1001/archneur.1987.00520220036012 3632374

[12] Roberts RO, Christianson TJ, Kremers WK, Mielke MM, Machulda MM, Vassilaki M, Petersen RC (2016) Association between olfactory dysfunction and amnestic mild cognitive impairment and Alzheimer disease dementia. JAMA Neurol 73:93–101 10.1001/jamaneurol.2015.2952 PMC4710557 26569387

[13] Croy I, Symmank A, Schellong J, Hummel C, Gerber J, Joraschky P, Hummel T (2014) Olfaction as a marker for depression in humans. J Affect Disord 160:80–86 10.1016/j.jad.2013.12.026 24445134

[14] Pabel LD, Hummel T, Weidner K, Croy I (2018) The impact of severity, course and duration of depression on olfactory function. J Affect Disord 238:194–203 10.1016/j.jad.2018.05.033 29886199

[15] Sabiniewicz A, Hoffmann L, Haehner A, Hummel T (2022) Symptoms of depression change with olfactory function. Sci Rep 12:5656 10.1038/s41598-022-09650-7 PMC8983665 35383250

[16] Doty RL, Shaman P, Dann M (1984) Development of the University of Pennsylvania Smell Identification Test: a standardized microencapsulated test of olfactory function. Physiol Behav 32:489–502 10.1016/0031-9384(84)90269-5 6463130

[17] Hummel T, Kobal G, Gudziol H, Mackay-Sim AJEA (2007) Normative data for the Sniffin’ Sticks including tests of odor identification, odor discrimination, and olfactory thresholds: an upgrade based on a group of more than 3,000 subjects. Eur Arch Otorhinolaryngol 264:237–243 10.1007/s00405-006-0173-0 17021776

[18] Hummel T, Liu DT, Müller CA, Stuck BA, Welge-Lüssen A, Hähner A (2023) Olfactory dysfunction: etiology, diagnosis, and treatment. Deutsches Ärzteblatt international 120:146 10.3238/arztebl.m2022.0411 PMC10198165 36647581

[20] Hummel T, Pfetzing U, Lötsch J (2010) A short olfactory test based on the identification of three odors. J Neurol 257:1316–1321 10.1007/s00415-010-5516-5 20232208

[21] Haehner A, Mayer AM, Landis BN, Pournaras I, Lill K, Gudziol V, Hummel T (2009) High test–retest reliability of the extended version of the Sniffin’Sticks test. Chem Senses 34:705–711 10.1093/chemse/bjp057 19759361

[22] Larsson M, Nilsson LG, Olofsson JK, Nordin S (2004) Demographic and cognitive predictors of cued odor identification: evidence from a population-based study. Chem Senses 29:547–554 10.1093/chemse/bjh059 15269128

[23] Sorokowska A, Sorokowski P, Hummel T (2014) Cross-cultural administration of an odor discrimination test. Chemosens Percept 7:85–90 10.1007/s12078-014-9169-0 PMC4037584 24883170

[24] Oleszkiewicz A, Schriever VA, Croy I, Haehner A, Hummel T (2019) Updated Sniffin’ sticks normative data based on an extended sample of 9139 subjects. Eur Arch Otorhinolaryngol 276:719–728 10.1007/s00405-018-5248-1 PMC6411676 30554358

[25] Gudziol V, Hummel T (2009) The influence of distractors on odor identification. Archives Otolaryngology–Head Neck Surg 135:143–145 10.1001/archotol.135.2.143 19221241

[26] Bland JM, Altman DG (1999) Measuring agreement in method comparison studies. Stat Methods Med Res 8:135–160 10.1177/096228029900800204 10501650

[27] Hummel T, Rosenheim K, Konnerth CG, Kobal G (2001) Screening of olfactory function with a four-minute odor identification test: reliability, normative data, and investigations in patients with olfactory loss. Ann Otorhinolaryngol 110:976–981 10.1177/000348940111001015 11642433

[28] Doty RL, McKeown DA, Lee WW, Shaman P (1995) A study of the test-retest reliability of ten olfactory tests. Chem Senses 20:645–656 10.1093/chemse/20.6.645 8788098

[29] Doty RL, Kamath V (2014) The influences of age on olfaction: a review. Front Psychol 5:20 10.3389/fpsyg.2014.00020 PMC3916729 24570664

[30] Sorokowski P, Karwowski M, Misiak M, Marczak MK, Dziekan M, Hummel T, Sorokowska A (2019) Sex differences in human olfaction: a meta-analysis. Front Psychol 10:242 10.3389/fpsyg.2019.00242 PMC6381007 30814965

[31] Croy I, Zehner C, Larsson M, Zucco GM, Hummel T (2015) Test–retest reliability and validity of the sniffin’TOM Odor Memory Test. Chem Senses 40:173–179 10.1093/chemse/bju069 25550307

[32] Sabiniewicz A, Wittig S, Haehner A, Müller C, Galvao C, Nakanishi M, Hummel T (2024) The digital scent device 20: an automated, self-administered odor identification test. Eur Arch Otorhinolaryngol 281:1–8 10.1007/s00405-024-08887-4 PMC11564298 39210076

