# Supplementary material for: A new framework for the labeling of odors in the Sniffin’ Sticks odor identification test: relevance of familiarity and test–retest assessment
Source: Eur Arch Otorhinolaryngol. 2026 Jun 15;283(9):6029–35. doi: 10.1007/s00405-026-10379-6 (PMC13614980; doi:10.1007/s00405-026-10379-6)
Supplement: Supplementary file 1 [file 405_2026_10379_MOESM1_ESM.docx]

Supplementary Table 1. Modified list of descriptors and identification rate of the target labels in the final version of SS-OIT.

Blue font color stands for Study 2, green for Study 3 and yellow for Study 4. Target labels are marked in yellow.

| 1 | orange | 95% | blackberry | 5% | strawberry | 0% | pineapple | 0% |
| --- | --- | --- | --- | --- | --- | --- | --- | --- |
| 2 | smoke | 0% | shoeleather | 95% | coffee | 0% | grass | 5% |
| 3 | aniseed | 0% | spruce | 5% | cinnamon | 95% | onion | 0% |
| 4 | chives | 0% | ginger | 5% | spruce | 10% | peppermint | 86% |
| 5 | coconut | 0% | blackberry | 10% | chocolate | 0% | banana | 90% |
| 6 | currant | 0% | apple | 0% | lemon | 100% | tomato | 0% |
| 7 | Cola | 0% | liquorice | 100% | chewing gum | 0% | cookies | 0% |
| 8 | paint thinner | 81% | rubber | 14% | onion | 0% | mustard | 5% |
| 9 | garlic | 95% | vinegar | 0% | rubber | 5% | carrot | 0% |
| 10 | cigarette | 0% | coffee | 100% | wine | 0% | glue | 0% |
| 11 | vanilla | 5% | peppermint | 0% | apple | 95% | chives | 0% |
| 12 | mustard | 0% | pepper | 0% | cinnamon | 0% | cloves | 100% |
| 13 | pear | 5% | plum | 5% | peach | 5% | pineapple | 86% |
| 14 | camomile | 10% | raspberry | 0% | rose | 86% | cherry | 5% |
| 15 | Rum | 10% | aniseed | 90% | coffee | 0% | spruce | 0% |
| 16 | fish | 100% | bread | 0% | cheese | 0% | wood | 0% |
| 17 | pear | 95% | carrot | 0% | mushroom | 0% | cloves | 5% |
| 18 | walnut | 0% | Cola | 95% | chewing gum | 5% | lemon | 0% |
| 19 | almonds | 0% | peppermint | 0% | lilac | 100% | chives | 0% |
| 20 | strawberry | 5% | grapes | 5% | peach | 10% | grapefruit | 81% |
| 21 | tomato | 14% | carrot | 5% | cheese | 0% | grass | 81% |
| 22 | lilac | 0% | plum | 0% | raspberry | 95% | melon | 5% |
| 23 | liquorice | 0% | eucalyptus | 100% | almonds | 0% | Rum | 0% |
| 24 | ginger | 81% | bell pepper | 5% | cloves | 10% | pepper | 5% |
| 25 | coconut | 95% | lilac | 0% | peanut | 0% | cinnamon | 5% |
| 26 | rose | 0% | lavender | 95% | grass | 0% | almonds | 5% |
| 27 | wood | 0% | glue | 5% | melon | 95% | pepper | 0% |
| 28 | cherry | 0% | strawberry | 5% | apple | 0% | peach | 95% |
| 29 | smoke | 14% | tomato | 0% | garlic | 0% | mushroom | 86% |
| 30 | shoeleather | 0% | cigarette | 0% | smoked meat | 95% | wood | 5% |
| 31 | peanut | 0% | caramel | 100% | cookies | 0% | honey | 0% |
| 32 | onion | 86% | mustard | 10% | bell pepper | 0% | nutmeg | 5% |
